# Polymer oxidation: A strategy for the controlled degradation of injectable cryogels

**DOI:** 10.1016/j.mtbio.2025.101743

**Published:** 2025-04-08

**Authors:** Alexandra Nukovic, Mohammad Hamrangsekachaee, Mahalakshmi Rajkumar, Gwyneth Wong, Emily R. Tressler, Sara M. Hashmi, Stephen M. Hatfield, Sidi A. Bencherif

**Affiliations:** a Department of Chemical Engineering, Northeastern University, Boston, MA, 02115, USA; b New England Inflammation and Tissue Protection Institute, Department of Pharmaceutical Sciences, Northeastern University, Boston, MA, 02115, USA; c Department of Bioengineering, Northeastern University, Boston, MA, 02115, USA; d Department of Biology, Northeastern University, Boston, MA, 02115, USA; e Department of Chemistry and Chemical Biology, Northeastern University, Boston, MA, 02115, USA; f Department of Mechanical and Industrial Engineering, Northeastern University, Boston, MA, 02115, USA; g Harvard John A. Paulson School of Engineering and Applied Sciences, Harvard University, Cambridge, MA, 02138, USA; h Polymers, Biopolymers, Surfaces Laboratory (PBS, UMR CNRS 6270), University of Rouen Normandy, 76130 Mont-Saint-Aignan, France

**Keywords:** Cryogel, Oxidation, Degradation, Hydrolysis, Biocompatibility

## Abstract

Cryogels, an advanced subclass of hydrogels, are widely used in biomedical applications such as tissue engineering, drug delivery, and immunotherapy. Biopolymers, like hyaluronic acid (HA), are key building blocks for cryogel fabrication due to their intrinsic biological properties, biocompatibility, and biodegradability. HA undergoes biodegradation through hydrolysis, enzymatic degradation, and oxidation, but becomes less susceptible to degradation once chemically modified. This modification is necessary for producing HA-based cryogels with unique properties, including an open macroporous network, mechanical resilience, shape memory, and syringe injectability. Endowing cryogels with resorbable features is essential for meeting regulatory requirements and improving treatment outcomes. To this end, HA was oxidized with sodium periodate (HA_ox_) and chemically modified with glycidyl methacrylate (HA_ox_GM) to create HA_ox_GM cryogels with controlled degradation. Oxidation of HA increased the susceptibility of the polymer backbone to breakdown through various mechanisms, including oxidative cleavage and alkaline hydrolysis. Compared to their poorly degradable counterparts, HA_ox_GM cryogels retained their advantageous properties despite reduced compressive strength. HA_ox_GM cryogels were highly cytocompatible, biocompatible, and tunable in degradation. When injected subcutaneously into mice, the HA_ox_GM cryogels were biocompatible and resorbed within two weeks. To validate the beneficial effect of controlled biodegradation in a relevant *in vivo* setting, we demonstrated that the degradation of HA_ox_GM cryogels accelerates ovalbumin release and enhances its uptake and response by immune cells in mice. This versatile oxidation strategy can be applied to a wide range of polymers, allowing better control over cryogel degradation, and advancing their potential for biomedical applications and clinical translation.

## Graphical abstract

**Figure:**
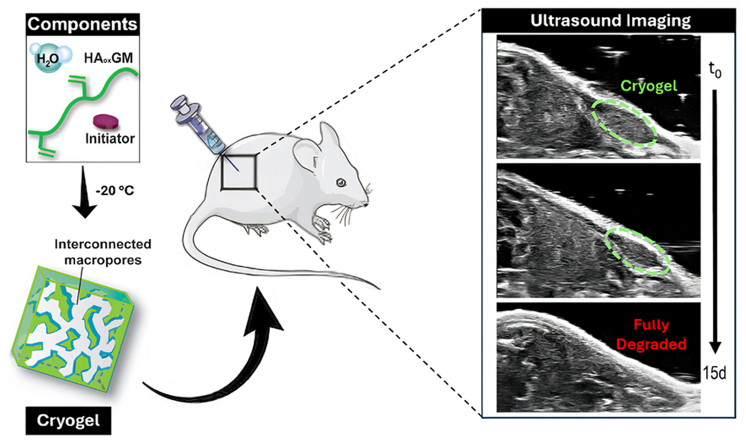

## 1 Introduction

Natural polymers, such as polysaccharides, are increasingly favored in biomaterial design for their excellent biocompatibility and ability to degrade safely within the body [1]. However, their degradation is often hindered by the slow hydrolysis of the polymer unless specifically engineered to target controlled degradation. Ideally, the degradation and resorption of these biomaterials should align with the timing of their application [2]. This approach not only minimizes long-term side effects but also eliminates the need for surgical removal [1,3,4].

Hyaluronic acid (HA), a polysaccharide glycosaminoglycan present in synovial fluids and the extracellular matrix (ECM), is widely used for various biomedical applications [[5], [6], [7], [8]]. HA is classified as a biodegradable polymer because it undergoes enzymatic and non-enzymatic degradation due to cellular activity *in vivo* [9]. Cells can produce hyaluronidase and reactive oxygen species (ROS) that mediate HA degradation [10,11]. Additionally, HA is susceptible to acidic and alkaline hydrolysis [9]. Effective degradation occurs through chain scissions of the polymer, breaking it into smaller, more manageable fragments that are more easily absorbed or eliminated by the body. This process is crucial for biomedical applications, as the controlled degradation of polysaccharides can significantly influence their effectiveness, including the controlled release of therapeutics and the promotion of tissue regeneration or formation [1,2].

Cryogels, a subclass of polymeric hydrogels, have gained significant attention in the biomedical field due to their unique properties, such as high elasticity, an open and interconnected macroporous network, shape memory, and syringe injectability [12,13]. These characteristics make cryogels increasingly popular for a variety of biomedical applications, including tissue engineering [[14], [15], [16], [17]], 3D cell culture [18,19], wound healing [20,21], drug delivery [22,23], and immunotherapy [[23], [24], [25], [26], [27], [28]]. HA-based cryogels, in particular, are widely used because of their excellent biocompatibility and inherent bioactivity. However, polysaccharides like HA often exhibit weak mechanical properties, which can limit their effectiveness. These properties can be enhanced through chemical modification [29]. For instance, introducing polymerizable groups, such as methacryloyl residues, into the polymer enables free radical polymerization and covalent crosslinking at sub-zero temperatures, resulting in the formation of cryogels [18,30,31]. Cryogels offer two main advantages as a delivery device: syringe injectability, which allows for minimally invasive implantation, and porous architecture, enhancing the release or exposure of cargo to infiltrating cells [12,32]. However, methacrylated HA-based gel systems exhibit slow degradation both *in vitro* and *in vivo* [7,33]. Therefore, controlling the degradability of these cryogels is a critical design consideration for optimizing their clinical applicability [34].

Various gelation strategies and crosslinking degrees have been explored to regulate the hydrolytic degradation of HA-based hydrogels and cryogels. However, these approaches not only influence degradation rates but also significantly impact the structural properties [1,35,36]. Oxidation strategies, including the use of sodium periodate (NaIO_4_), have been investigated to facilitate the degradation of poorly degradable polysaccharides such as HA *in vivo* [[37], [38], [39], [40], [41], [42]]. In HA, NaIO_4_ selectively oxidizes vicinal diols, breaking the carbon rings and converting alcohol groups into aldehydes [43]. This transformation introduces a hydrolytically liable open-chain adduct into the polymer backbone, inducing primarily beta-elimination via alkaline hydrolysis and significantly accelerating polymer degradation [37]. In this work, we hypothesized that tailored NaIO_4_ oxidation of HA could enable controlled degradation of mechanically robust cryogels, thereby enhancing their performance for therapeutic applications. To test this, we oxidized HA (HA_ox_) prior to glycidyl methacrylation (HA_ox_GM) and characterized the degree of oxidation (DO), degree of methacrylation (DM), viscosity, and final molecular weight of the modified polymer. Subsequently, HA_ox_GM cryogels were fabricated and evaluated for their syringe injectability, pore connectivity, swelling, compressive strength, and macroporous network. The degradation kinetics of HA_ox_GM cryogels were then studied under varying DO levels and environmental conditions, such as pH and exposure to hyaluronidase, an enzyme that degrades HA. To assess their performance further, the cytocompatibility and biocompatibility of HA_ox_GM cryogels were evaluated both *in vitro* and *in vivo*. The cryogels were also subcutaneously injected into mice to monitor their biodegradation using ultrasound imaging. Finally, to investigate the effect of controlled biodegradation in a relevant *in vivo* setting, we tested whether the degradation of HA_ox_GM cryogels could accelerate the release of the model protein ovalbumin (OVA) and enhance its uptake by immune cells in mice.

## 2 Materials and methods

### 2.1 Materials

HA sodium salt (∼1.5 MDa) was purchased from Glentham Life Sciences (Corsham, UK). NaIO_4_, Dulbecco's phosphate buffered saline (PBS), sodium bicarbonate (NaHCO_3_), 2-(N-morpholino)ethanesulfonic acid (MES hydrate, C_6_H_13_NO_4_S · xH_2_O), sodium hydroxide (NaOH), glycidyl methacrylate (GM), N, N-dimethylformamide (DMF), triethylamine (TEA), tetramethylethylenediamine (TEMED), ammonium persulfate (APS), N-(3-dimethylaminopropyl)-N′-ethylcarbodiimide (EDC), N-hydroxysuccinimide (NHS), paraformaldehyde (PFA), Triton X-100, and 4′,6-diamidino-2-phenylindole (DAPI), fetal bovine serum, and hyaluronidase from bovine testes (Type IV-S) were acquired from MilliporeSigma (St. Louis, MO, USA). Hydroxylamine hydrochloride, Acetate buffer, deuterium oxide (D_2_O), penicillin/streptomycin, pHrodo^TM^ Red, SE, BODIPY^TM^ FL, SE, and eBioscience^TM^ Fixable Viability Dye eFluor^TM^ 506 were purchased from Thermo Fisher Scientific (Waltham, MA, USA). ViaQuant Fixable Far-Red Dead Cell Staining Kit and Acti-Stain^TM^ 488 were obtained from Genecopoeia (Rockville, MD, USA) and Cytoskeleton, Inc (Denver, CO, USA), respectively. The National Institutes of Health (NIH) 3T3 cells were purchased from American Type Culture Collection (Manassas, VA, USA). Recombinant murine granulocyte-macrophage colony-stimulating factor (mGM-CSF), Dulbecco's Modified Eagle Medium (DMEM), and Roswell Park Memorial Institute 1640 medium (RPMI) were procured from Gibco (Waltham, MA, USA). Lipopolysaccharide (LPS-EB) derived from *Escherichia (E.) coli* and *EndoFit*^*TM*^ *OVA* were purchased from InvivoGen (San Diego, CA, USA). Amine-terminated GGGGRGDSP (G_4_RGDSP) peptide was ordered from Peptide 2.0 (Chantilly, VA, USA). Acrylate-polyethylene glycol (PEG)-succinimidyl (AP-NHS, 3.4 kDa) was purchased from Laysan Bio (Arab, AL, USA).

### 2.2 Synthesis of HA_ox_GM

#### 2.2.1 Oxidation of HA

First, HA (5 mg/mL) was dissolved in 200 mL of deionized water (diH_2_O). Next, NaIO_4_ was added to the polymer solution based on the molar ratio of NaIO_4_ to HA for the required degree of oxidation (DO = 5–40 %). To target a 40 % oxidation, 213.3 mg of NaIO_4_ was used (Table S1). The reaction was conducted light-free under stirring at room temperature (RT) for 24 h. Next, ethylene glycol was added in excess to react with any unreacted NaIO_4_ for 1 h. Finally, oxidized HA (HA_ox_) was dialyzed with a membrane having a molecular weight cutoff (MWCO) of 10 kDa against diH_2_O for 3 d, lyophilized, and then stored at −20 °C until further use.

#### 2.2.2 Methacrylation of HA and HA_ox_

The methacrylation of HA and HA_ox_ was performed as previously reported [12,29]. Briefly, HA or HA_ox_ and GM were first dissolved at a molar ratio of 1:50 (HA:GM or HA_ox_:GM) in a co-solvent mixture (PBS and DMF). For the modification of HA, 200 mg of HA was dissolved in 40 mL of PBS. Next, under stirring, 13.5 mL of DMF, 3.5 g of GM, and 1.8 g of TEA were slowly added to the reaction mixture. The reaction was conducted light-free at RT for 5 d. Finally, glycidyl methacrylated HA or HA_ox_ (HAGM or HA_ox_GM) was precipitated in cold acetone, vacuum dried at RT overnight, and then stored at −20 °C until further use.

#### 2.2.3 Synthesis of acrylate-PEG-G_4_RGDSP (APR)

APR was synthesized by coupling amine-terminated G_4_RGDSP to AP-NHS comonomer (molar ratio 1:1). The reaction proceeded for 4 h in NaHCO_3_ buffer (pH 8.5) at RT and the solution was subsequently freeze-dried to obtain APR. The product was then stored at −20 °C until further use.

### 2.3 Characterization of HAGM and HA_ox_GM

#### 2.3.1 Assessment of DO

The presence of aldehydes in HA_ox_ and HA_ox_GM was confirmed using Attenuated Total Reflectance Fourier-transform spectroscopy (ATR-FTIR) with a Bruker Vertex 70 FT-IR (Bruker, Billerica, MA, USA) and analyzed with OriginLab software (OriginLab, Northampton, MA, USA). The DO was characterized by determining aldehyde content using a previously reported hydroxylamine hydrochloride method [44,45]. Briefly, HA_ox_ was dissolved and reacted for 24 h in a 0.2 M hydroxylamine hydrochloride solution at pH 3.5. After the reaction, the pH was adjusted back to 3.5 using NaOH, and the titration volume was used to calculate the quantity (in moles) of aldehydes that reacted. The DO was then calculated as the ratio of consumed aldehydes to HA units, as depicted in Equation (1).

(1) $$DO\left(\%\right)=\frac{\left(V_{HAox}-V_{HA}\right)\times C_{NaOH}}{m_{polymer}}\times 100$$

V_HAox_ and V_HA_ represent the volumes of NaOH (mL) used to titrate the solution to pH 3.5, C_NaOH_ is the concentration of NaOH solution (mol/mL), and m_polymer_ is the quantity of HA or HA_ox_ (mol).

#### 2.3.2 Calculation of DM

Proton nuclear magnetic resonance (^1^H NMR) spectroscopy was used to evaluate the DM of HAGM and HA_ox_GM using a Varian Inova-500 NMR spectrometer (Agilent, Santa Clara, CA, USA). D_2_O was used as the solvent, and the concentration of the modified polymers was kept at 1 % (w/v). All ^1^H NMR spectra were obtained at RT, 15 Hz sample spinning, 45° tip angle for the observation pulse, and an 8 μs recycle delay, for 128 scans. The relative peak integrations of methacryloyl groups (δ5.0–6.0 ppm) were correlated to the integration of the methyl group of HA (δ1.5–2.0 ppm). Peak areas were integrated using Topspin (Bruker, Billerica, MA, USA) NMR analysis software, and the DMs for HAGM and HA_ox_GM were determined as previously described [12,29].

#### 2.3.3 Viscosity measurements

The viscosity of HA, HAGM, HA_ox_, and HA_ox_GM in diH_2_O was evaluated by cone-plate rheometry (40 mm diameter, cone angle 1.00778°) using a Discovery HR-3 rheometer (TA Instruments, New Castle, DE, USA). Polymer solutions (0.5 % w/v) of HA, HAGM, HA_ox_, and HA_ox_GM in diH_2_O were analyzed. A flow sweep procedure was run at 25 °C with a shear rate (s^−^^1^) from 10^−2^ to 10^3,^ and the stress (Pa) and viscosity (Pa.s) were measured.

#### 2.3.4 Molecular weight characterization

The molar mass moments (kDa) of HA_ox_ (DO 1 %), HAGM, and HA_ox_GM (DO 1 %) were determined using gel permeation chromatography with multi-angle light scattering detection (GPC-MALS) (Cambridge Polymer Group, Woburn, MA, USA). Polymer solutions (1 mg/mL) were prepared in water containing 0.1 M sodium dihydrogen phosphate and 0.05 % sodium azide and filtered through 0.45 μm polyethersulfone syringe filters. Samples were analyzed using Agilent PL aquagel-OH Mixed-H and Agilent PL aquagel-OH 60 (300 mm x 7.5 mm, 8 μm) separation columns (Agilent, Santa Clara, CA, USA). Molar mass moments (M_n_, M_p_, M_w_, M_z_) and polydispersity of each sample were characterized through refractive index detection and light scattering data analysis using Wyatt ASTRA software.

### 2.4 Fabrication of cryogels

Cryogels were fabricated via free radical polymerization with either HAGM or HA_ox_GM (4 % w/v) in diH_2_O at subzero temperature using a redox initiator system (TEMED and APS). HAGM cryogels required 0.07 % and 0.28 % w/v, whereas for HA_ox_GM, 0.14 % and 0.56 % w/v were used for TEMED and APS, respectively. The cryogels were formed either in cuboidal (4 x 4 x 1 mm^3^) or cylindrical (height x diameter: 6 x 8 mm^3^) Teflon molds. The molds were placed in a freezer at −20 °C for 20 h to allow cryopolymerization. Finally, cryogels were thawed at RT, removed from the molds, washed in PBS, and immediately used for all subsequent experiments. For cell studies, APR (0.8 % w/v) was also used as a co-monomer to promote cell-matrix interactions.

### 2.5 Physical characterization of cryogels

#### 2.5.1 Pore size, pore connectivity, and swelling ratio

To determine the pore size and pore size distribution by scanning electron microscopy, lyophilized cuboidal cryogels (4 x 4 x 1 mm^3^) were mounted on the sample holder using carbon tape and sputter-coated with platinum/palladium up to 5 nm thickness. Samples were then imaged using secondary electron detection on a Zeiss Supra 25 scanning electron microscope (Zeiss, Oberkochen, Germany) while operating at 3 kV and 10 mA. The average pore sizes (μm) and pore distributions were quantified by measuring pore diameters using Fiji: ImageJ.

To quantify the degrees of pore connectivity, fully hydrated cylindrical cryogels (6 x 8 mm^3^) were first weighed on an analytical scale (W_s_). Next, a Kimwipe was lightly applied to the cryogels’ surfaces to wick away free water. The weight of partially dehydrated cryogels was recorded (W_w_). The degree of pore connectivity was calculated based on the weight of water wicked away (W_s_ – W_w_) divided by the initial weight of fully hydrated cryogels (W_s_) as depicted in Equation (2).

(2) $$Pore\;connectivity\;\left(\%\right)=\frac{\left(W_{s}-W_{w}\right)}{W_{s}}\times 100$$

The swelling ratio was measured using a conventional gravimetric procedure. Cylindrical cryogels (6 x 8 mm^3^) were prepared and soaked in PBS for 5 min and 24 h prior to the experiment. The equilibrated mass swelling ratio (Q_M_) was calculated by dividing the mass of fully swollen (W_wet_) by the mass of freeze-dried cryogel (W_dry_), as depicted in Equation (3). The cryogels were washed in diH_2_O for salt removal prior to being freeze-dried.

(3) $$Q_{M}=\frac{W_{wet}}{W_{dry}}$$

#### 2.5.2 Mechanical properties and syringe injectability

Young's modulus of cryogels were determined using a TA Electroforce 5500 mechanical loading device (TA Instruments, New Castle, DE, USA). Cylindrical cryogels (6 x 8 mm^3^) were dynamically compressed between two parallel plates at a rate of 0.01 mm/s until a displacement of 50 % of the sample height. Load (N) and displacement (mm) were recorded and used to plot stress *vs.* strain. The Young's modulus was defined as the slope of the stress-strain curve. Cryogels were kept hydrated in PBS (pH 7.4) throughout the tests.

To test syringe injectability and shape memory, cuboidal cryogels (4 × 4 × 1 mm^3^) were placed at the aperture of a 16G hypodermic needle. The needle was then inserted into a 1 mL syringe prefilled with 0.2 mL of PBS, and the cryogels were subsequently injected. Videos of cryogel injections were recorded, and the injection shear rate (s^−^^1^) was estimated as depicted in Equation (4).

(4) $$Shear\;rate\;\left(s^{‐1}\right)=\frac{needle\;length\;\left(mm\right)}{needle\;radius\;\left(mm\right)\times plunge\;time\;\left(s\right)}$$

#### 2.5.3 Degradation of HA_ox_GM cryogels

To assess the degradation kinetics under various conditions, cuboidal cryogels (4 × 4 × 1 mm^3^) were incubated at 37 °C under orbital shaking in various buffer solutions (pH 3–8.5) or in PBS (pH 7.4). Enzymatic degradation was investigated using MES buffer (pH 5.35) supplemented with hyaluronidase from bovine testes (25 mg/mL, 750–3000 U/mg). The buffer pH was selected based on the manufacturer's recommendation, and the enzyme concentration was optimized to achieve substantial degradation over 2 weeks. All degradation buffers were replenished every other day throughout the study. Degradation rates were obtained by performing a linear regression of the mass loss (%) as a function of time (d). At each time point, the cryogels were washed in diH_2_O to remove salts, lyophilized, and their average dry weight was measured.

### 2.6 Cytocompatibility and biocompatibility of HA_ox_GM cryogels

#### 2.6.1 Cytocompatibility

NIH 3T3 fibroblast cells were cultured in DMEM supplemented with FBS (10 % v/v) and penicillin/streptomycin (1 % v/v). RGD-containing cuboidal cryogels (4 × 4 × 1 mm^3^) were briefly sterilized using 70 % ethanol, followed by multiple washes with PBS. Next, cells were seeded onto the cryogels at a concentration of 2x10^5^ cells/cryogel and subsequently incubated at 37 °C in a humidified atmosphere containing 5 % CO_2_. Cell assessment was performed 24 h post-seeding. After fixation (4 % PFA) and permeabilization (0.1 % Triton X-100), cells were stained with Acti-Stain^TM^ and DAPI to visualize the cytoskeleton and nuclei, respectively. Cell viability was determined using a ViaQuant Fixable Far-Red Dead Cell Staining Kit according to the manufacturer's instructions. Fluorescence images were acquired using a Zeiss LSM 800 confocal microscope (Zeiss, Oberkochen, Germany) and analyzed using ImageJ software. Fractions of cell viability were determined as the ratio of viable cells to the total number of cells.

#### 2.6.2 Generation and *in vitro* activation assay of bone marrow-derived dendritic cells (BMDCs)

Dendritic cell (DC) activation studies were performed using BMDCs isolated from 6–8-week-old female C57BL/6 mice (The Jackson Laboratory, Bar Harbor, ME, USA). The femurs and tibialis of mice were explanted, and the bone marrow was flushed with PBS (2 mL, 27G needle). Next, cells were dissociated by pipetting, centrifuged (5 min, 300 g), and resuspended (10^6^ cells/mL) in RPMI supplemented with 10 % heat-inactivated FBS, 100 U/mL penicillin, 100 μg/mL streptomycin, 2 mM l-glutamine, 50 μM 2-mercaptoethanol, and 20 ng/mL mGM-CSF. At day 5, the BMDCs were resuspended (10^6^ cells/mL) in complete RPMI with 10 ng/mL mGM-CSF. At day 10, BMDCs were collected and used.

To evaluate the response of BMDCs to different types of cryogels, sterilized cuboidal cryogels (4 x 4 x 1 mm^3^) were incubated with BMDCs for 24 h in RPMI 1640 supplemented with 10 % FBS and 1 % penicillin-streptomycin. BMDC activation and maturation were subsequently evaluated by flow cytometry (AttuneNxT, Thermo Fisher Scientific) using the following fluorescent antibodies (Biolegend, San Diego, CA, USA): PE/Cyanine 7-conjugated anti-mouse CD11c (clone N418), APC/Cyanine 7- conjugated anti-mouse I-A/I-E (clone M5/114.15.2), Alexa Fluor 700-conjugated anti-mouse CD86 (clone GL-1), Alexa Fluor 488-conjugated anti-mouse CD40 (clone HM40-3), and eBioscience^TM^ Fixable Viability Dye eFluor 506. In addition, the culture supernatant was collected, and the concentration of proinflammatory cytokines (IL-6, TNF-α, and IL-12p70) was quantified using LEGENDplex^TM^ multiplexing beads. Negative and positive controls consisted of BMDCs cultured in media alone and media supplemented with 250 ng/mL of LPS, respectively.

#### 2.6.3 Biocompatibility

The *in vivo* response and tissue integration were evaluated following subcutaneous injection of HAGM and HA_ox_GM (DO 10 %) cryogels into mice. Cryogels were sterilized briefly with 70 % ethanol, followed by successive washes with sterile PBS. Each cryogel, suspended in 0.2 mL of PBS, was injected subcutaneously into both dorsal flanks of eight-week-old female C57BL/6J mice (The Jackson Laboratory, Bar Harbor, ME, USA, n = 5) using a 16G hypodermic needle. After 7 d post-injection, mice were sacrificed to assess cellular infiltration, tissue integration, and any signs of inflammation. Cryogels, along with the surrounding tissues, were excised, fixed in 4 % PFA for 48 h, embedded in paraffin, cryosectioned into 5-μm-thick slices, and stained with hematoxylin and eosin (H&E) or Masson's trichrome (MT) for standard histological evaluation (iHisto, Salem, MA, USA).

#### *2.6.4* *In vivo* degradation

Sterile HAGM and HA_ox_GM (DO 10 %) cryogels were subcutaneously injected into the dorsal flanks of eight-week-old female C57BL/6J mice as described above. Over the course of two weeks, the mice were anesthetized at various time points, their fur around the injection site was removed using Nair hair removal lotion, and a generic ultrasound gel (Aquasonic, Parker Laboratories, Fairfield, NJ, USA) was applied to the skin. Subsequently, 3D imaging of the implanted cryogels were acquired using a 29-15 MHz (UHF29x) linear probe ultrasound (VisualSonics Vevo F2). The volumes of the visualized implanted cryogels were monitored and calculated using VevoLab software (FUJIFILM VisualSonics, Toronto, ON, CA).

### 2.7 Delivery of OVA to immune cells

#### *2.7.1* *In vitro* release of OVA from cryogels

Pre-formed HAGM and HA_ox_GM (DO 10 %) cryogels were reacted with 10 μg of OVA per cryogel using EDC/NHS coupling chemistry. Cryogels were incubated in PBS at 4 °C under orbital shaking. Every few days over a 2-week period, the supernatant was collected, and the concentration of released OVA was quantified using Micro-BCA analysis (Thermo Fisher, Waltham, MA, USA).

#### *2.7.2* *In vivo* degradation-supported delivery of OVA to immune cells

To evaluate the degradation of protein-loaded cryogels and the associated immune cell phagocytosis, OVA was labeled with pHrodo^TM^ Red and BODIPY^TM^ FL (pHrodo-BODIPY-OVA) via NHS-mediated coupling reactions. Briefly, pHrodo^TM^ Red-SE and BODIPY^TM^ FL-SE were mixed with OVA at molar ratios of 20:1 and 30:1, respectively, in 0.1 M sodium bicarbonate buffer (pH 8.4) and allowed to react for 2 h at RT, protected from light. The labeled OVA was purified using Pierce^TM^ dye removal columns. Pre-formed HAGM and HA_ox_GM (DO 10 %) cryogels were then reacted with 10 μg of pHrodo-BODIPY-OVA per cryogel using EDC/NHS coupling chemistry. The cryogels were sterilized with 70 % ethanol, followed by successive sterile PBS washes to remove unreacted reagents. Next, the fluorescently labeled OVA-loaded cryogels were subcutaneously injected into both dorsal flanks of eight-week-old female C57BL/6J mice along mGM-CSF (1.5 μg/cryogel) supplemented PBS. Controls included bolus delivery of pHrodo-BODIPY-OVA and unloaded “blank” HAGM cryogels. Cryogel degradation was monitored using ultrasound imaging. After 1 week, the mice were euthanized, and cryogels along with inguinal lymph nodes were harvested. The samples were homogenized over a 40 μm cell strainer to create single-cell suspensions, which were then washed and stained for flow cytometry analysis using the AttuneNxT flow cytometer (Thermo Fisher Scientific). The following fluorescent antibodies (Biolegend, San Diego, CA, USA) were used for staining: Brilliant Violet 421-conjugated anti-mouse CD11c (clone N418), Brilliant Violet 605-conjugated CD19 (clone 6D5), APC-conjugated F4/80 (clone BM8), APC/Cyanine 7- conjugated anti-mouse I-A/I-E (clone M5/114.15.2), PerCP/Cy 5.5-conjugated anti-mouse CD45 (clone S18009F), and eBioscience^TM^ Fixable Viability Dye eFluor 506.

### 2.8 Regulatory compliance and approval for animal studies

All experiments requiring mice were conducted in compliance with the National Institutes of Health (NIH) guidelines and approved by the Division of Laboratory Animal Medicine (DLAM) and Northeastern University Institutional Animal Care and Use Committee (protocol number 23–0512R).

### 2.9 Statistical analysis

All data were presented as mean ± standard error of the mean (SEM). Statistical analyses were performed using GraphPad Prism (La Jolla, CA, USA). Significant differences between groups were analyzed by Welch's *t*-test, one-way analysis of variance (ANOVA), and Tukey's post-hoc test. Differences were considered statistically significant at ∗p < 0.05, ∗∗p < 0.01, ∗∗∗p < 0.001, and ∗∗∗∗p < 0.0001.

## 3 Results

### 3.1 Synthesis and characterization of HA_ox_GM

**Fig. 1:**
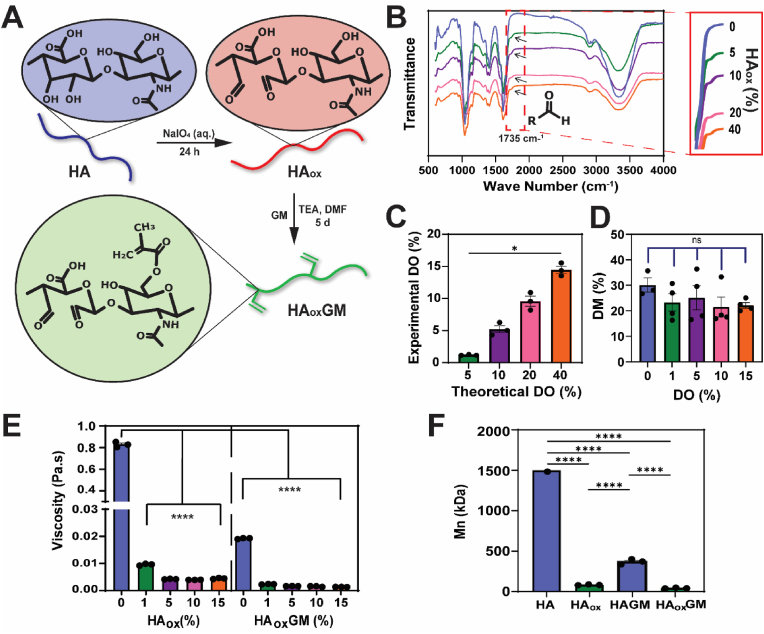
**Synthesis and characterization of HA_ox_GM. A**) Overview of the chemical synthesis of HA_ox_GM. HA is oxidized with NaIO_4_ and subsequently methacrylated with GM. **B**) ATR-FTIR of HA_ox_ with varying DOs (DO = 0–40 %). The characteristic aldehyde peaks are observed at 1725 cm^−1^ (shown in the inset). **C**) Comparison of experimental and theoretical DO for HA_ox_. **D**) DM of HA_ox_GM quantified using ^1^H NMR spectroscopy. **E**) Viscosity measurements of HA_ox_ and HA_ox_GM in diH_2_O (0.5 % w/v) at shear rate of 10 s^−1^. **F**) Number average molecular weight (M_n_) of HA, HA_ox_ (DO = 1 %), HAGM, and HA_ox_GM (DO = 1 %). Data are presented as mean ± SEM (n = 3–4). Statistical analysis was performed using one-way ANOVA and Tukey's post hoc test: ns (not significant) ≥ 0.05, ∗p < 0.05, ∗∗∗∗p < 0.0001.

To design injectable cryogels with controlled degradation, HA was partially oxidized with NaIO_4_ before methacrylation to facilitate alkaline hydrolysis (Fig. 1A). The efficiency of the oxidation reaction was assessed by confirming the presence of aldehyde residues on the polymer chain using ATR-FTIR analysis. Unlike unoxidized HA, a characteristic aldehyde peak for C = O stretching at 1735 cm^−1^ was observed across all HA_ox_ samples (theoretical DO = 5, 10, 20, and 40 %), as depicted in Fig. 1B. This indicates that vicinal diols in HA were cleaved during oxidation, forming aldehyde groups at the sites of bond cleavage.

The DO was quantified using hydroxylamine hydrochloride titration, where it was calculated based on the ratio of aldehydes to HA repeat units. Experimental results showed that the measured DOs were two- or threefold lower than the theoretical values, with the reaction efficiency decreasing as NaIO_4_ concentration increased (Fig. 1C). Subsequently, HA_ox_ was reacted with GM to introduce crosslinkable groups, and the DM was quantified by ^1^H NMR. The DM in HA_ox_GM (DO = 1, 5, 10, and 15 %) was ∼23 % for all oxidized polymers, slightly lower than ∼30 % observed in unoxidized HAGM (DO = 0 %). However, the differences were not statistically significant between the groups (Fig. 1D, Fig. S1). ATR-FTIR confirmed that aldehyde groups were retained after methacrylation (Fig. S2).

HA_ox_GM exhibited a significant decrease in viscosity compared to HAGM, an important consideration for polymer handling and cryopolymerization. To understand this reduction, rheological measurements were performed to evaluate the viscosity of polymer solutions (0.5 % w/v) as a function of shear rate (s^−^^1^) before and after oxidation and methacrylation independently. Flow curves revealed that HA solutions exhibited highly viscous, non-Newtonian behavior, with a low shear plateau viscosity of ∼3 Pa.s, dropping only beyond a shear rate of 1 s^−1^. In contrast, HA_ox_, HAGM, and HA_ox_GM solutions (DO = 1, 5, 10, and 15 %) displayed Newtonian behavior, characterized by lower viscosity (<0.1 Pa.s) that remained constant regardless of shear rate (Fig. S3). A comparison of solution viscosity was done at 10 s^−^^1^, where the Newtonian polymer solutions showed stable readings. HAGM displayed a nearly 40-fold decrease in viscosity compared to HA, with values of 0.019 ± 3.0 × 10^−^^5^ Pa.s and 0.83 ± 0.013 Pa.s, respectively. Additionally, all HA_ox_ variants demonstrated an over 80-fold reduction in viscosity compared to HA, with values of ≤0.0095 ± 1.3 × 10^−^^4^ Pa.s. This reduction in viscosity was further amplified after methacrylation (Fig. 1E). This transition in flow behavior at higher degrees of oxidation (DOs) is attributed to chain scission (i.e., breaking of glycosidic bonds) and partial degradation of HA during the chemical reactions. GPC-MALS analysis confirmed significant degradation of polymer chains. The starting HA polymer had a number average molecular weight (M_n_) of ∼1.5 MDa, while the M_n_ values of HA_ox_, HAGM, and HA_ox_GM were reduced to 83 ± 4 kDa, 370 ± 33 kDa, and 43 ± 5 kDa, respectively (Fig. 1F). Additional molecular weights and dispersity data are summarized in Table S2. Altogether, these findings indicate that partial oxidation of HA prior to methacrylation induces substantial chain scission, leading to decreased viscosity and a shift from non-Newtonian to Newtonian behavior. This alteration in solution properties also suggests potential changes in the mechanical integrity of the resulting cryogels. To investigate this further, subsequent studies assessed the mechanical integrity and injectability of HA_ox_GM cryogels.

### 3.2 Fabrication and characterization of HA_ox_GM cryogels

**Fig. 2:**
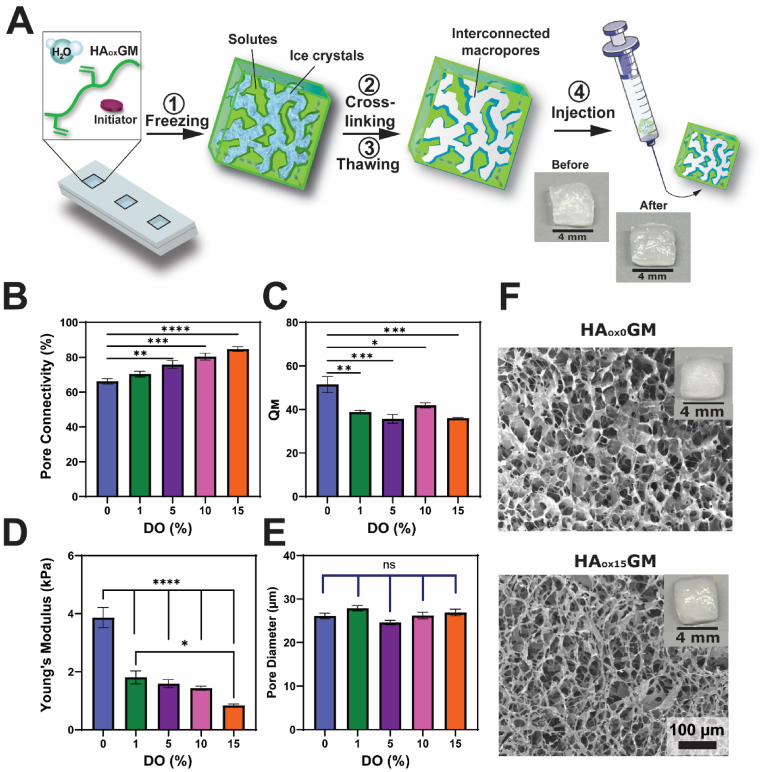
**Fabrication and physical properties of HA_ox_GM cryogels. A**) Schematic depicting the process of cryogelation and syringe injectability: HA_ox_GM is first dissolved in diH_2_O and mixed with a redox initiator system, frozen at −20 °C in a precooled mold for 20 h, and then thawed at RT, resulting in a macroporous and interconnected network suitable for syringe injection. **B–E**) Comparative analysis of pore connectivity, swelling ratio (Q_M_), Young's modulus, and pore diameter of HA_ox_GM (DO = 1–15 %) cryogels *vs.* unoxidized HAGM cryogels. **F**) Scanning electron microscopy images of HAGM and HA_ox_GM (DO = 15 %) cryogels. Data are presented as mean ± SEM (n = 3–4). Statistical analysis was performed using one-way ANOVA and Tukey's post hoc test: ns ≥ 0.05, ∗p < 0.05, ∗∗p < 0.01, ∗∗∗p < 0.001, and ∗∗∗∗p < 0.0001.

Cryogels were synthesized with HA_ox_GM and tested for their mechanical integrity and injectability (Fig. 2A). As expected, compared to HAGM, the lower molecular weight of HA_ox_GM significantly impacted the physical properties of the resulting cryogels. First, as described under the cryogel fabrication methods, the concentration of the redox initiator system (TEMED and APS) had to be doubled to form HA_ox_GM cryogels with a structure comparable to that of HAGM cryogels. Increasing the initiator amount likely helps compensate for the loss of molecular weight by promoting more effective crosslinking, regulating the polymerization rate, and partially preserving the desired physical and mechanical properties of the cryogels [46,47]. In addition, the injectability threshold through a 16G needle was set at 10 % oxidation, as injected cryogels formulated with HA_ox_GM oxidized to 15 % displayed partial fracture (Fig. S4, Videos S1–S5). The injection shear rate (s^−^^1^) was estimated from the time required to fully depress the syringe plunger for each cryogel. The calculated values were comparable in magnitude to those obtained from rheometric analysis (Table S3). Injection results indicated that cryogels polymerized from lower viscosity polymers allowed for faster injection times. Next, key physical properties of HA_ox_GM cryogels were evaluated, including pore connectivity, swelling ratio, compressive Young's modulus, and pore diameter. Pore connectivity increased with the DO, rising from 66 ± 1.6 in unoxidized cryogels (DO = 0 %) to 85 ± 1.4 for those with a 15 % DO (Fig. 2B). Additionally, the swelling ratio decreased as the DO increased, from Q_M_ = 51 ± 3.7 for unoxidized cryogels (DO = 0 %) to 39 ± 0.8, 36 ± 2.0, 42 ± 1.2, and 36 ± 0.3 for 1, 5, 10, and 15 % DO, respectively (Fig. 2C). Consistent with known cryogel properties, swelling occurred rapidly, with the cryogels reaching full hydration within minutes (Table S4). These findings are consistent with prior studies demonstrating that reduced molecular weight of the polymer increases pore connectivity, while increased crosslinking density decreases the swelling ratio [48,49]. The compressive Young's modulus of HA_ox_GM cryogels was significantly reduced compared to HAGM cryogels, further diminishing with increasing the DO. HA_ox_GM cryogels with 15 % DO exhibited a modulus of 0.84 ± 0.05 kPa, compared to 1.8 ± 0.22 kPa for 1 % DO (Fig. 2D). However, the cryogels' macroporous structure was maintained, with consistent pore diameters between 20 and 30 μm across all groups (Fig. 2E and F). Despite their reduced mechanical integrity, HA_ox_GM cryogels maintained syringe injectability and exhibited consistently large, highly interconnected pores.

Supplementary video related to this article can be found at https://doi.org/10.1016/j.mtbio.2025.101743

The following are the supplementary data related to this article:

Multimedia component 2 Multimedia component 2

Multimedia component 3 Multimedia component 3

Multimedia component 4 Multimedia component 4

Multimedia component 5 Multimedia component 5

Multimedia component 6 Multimedia component 6

### 3.3 Degradation mechanisms of cryogels

**Fig. 3:**
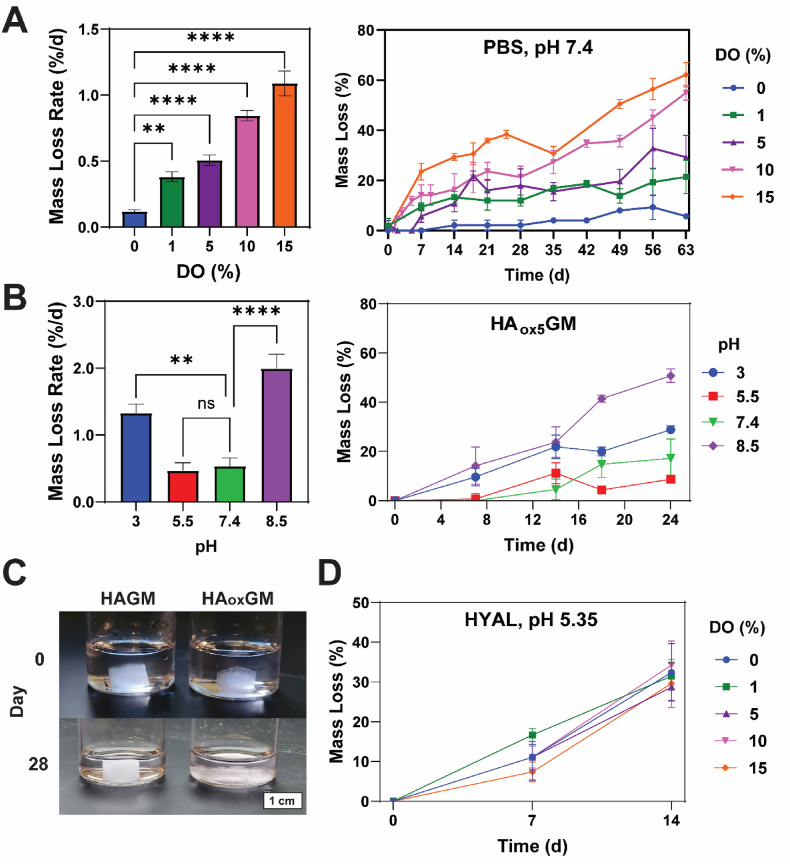
**HA_ox_GM cryogels exhibit enhanced degradation across various conditions. A**) Mass loss rate (%/d) and corresponding degradation kinetics of HA_ox_GM (DO = 0–15 %) cryogels incubated in PBS (pH 7.4) at 37 °C across various time points (0–63 d). **B**) Mass loss rate (%/d) and corresponding degradation kinetics of HA_ox_GM (i.e., DO = 5 %) cryogels incubated in different buffer solutions (pH 3–8.5) at 37 °C across various time points (0–24 d). **C**) Visual representation of the degradation of HAGM (non-oxidized: DO = 0 %) and HA_ox_GM (DO = 15 %) cryogels incubated in a sodium bicarbonate solution (pH 8.5) at 37 °C after 28 d. **D**) Degradation kinetics of HA_ox_GM (DO = 0–15 %) cryogels incubated in a buffer solution (pH 5.35) supplemented with hyaluronidase (HYAL) at 37 °C across various time points (0–14 d). Data are presented as mean ± SEM (n = 3–5). Statistical analysis was performed using one-way ANOVA: ns ≥ 0.05, ∗∗p < 0.01, ∗∗∗p < 0.001, and ∗∗∗∗p < 0.0001.

Compared to unoxidized HAGM, our oxidation strategy for HA_ox_GM resulted in significantly accelerated degradation of cryogels. Higher DOs increased the mass loss rate *in vitro* under physiological conditions (pH 7.4, 37 °C). For instance, HA_ox_GM (DO = 15 %) cryogels degraded at a rate of ∼1 %/d, with complete degradation estimated within 100 d (Fig. 3A). To assess the contribution of acidic and alkaline hydrolysis to degradation, tests were conducted under various pH conditions. In basic environments, the degradation of oxidized polysaccharides is primarily driven by aldehyde-induced carbanion formation, which facilitates β-hydrogen elimination and backbone cleavage, with additional contributions from alkali-catalyzed hydrolysis and deprotonation effects [50,51]. In acidic environments, degradation is primarily driven by acid-catalyzed hydrolysis, where protonation of functional groups (such as esters and aldehydes) increases the susceptibility of polymer backbone to cleavage [51]. HA_ox_GM (DO = 5 %) cryogels exhibited a 4-fold increase in degradation rate under basic conditions (pH 8.5) and 3-fold increase under acidic conditions (pH 3) compared to PBS (pH 7.4), likely due to alkaline and acid-catalyzed hydrolysis mechanisms (Fig. 3B). At pH 8.5 and 37 °C, HA_ox_GM (DO = 15 %) cryogels nearly fully degraded within 28 d, while HAGM cryogels (DO = 0 %) remained intact (Fig. 3C). Interestingly, oxidation did not impact enzymatic degradation, as all cryogels lost ∼30 % mass after 2 weeks when incubated with a large excess of hyaluronidase (Fig. 3D). These results highlight the multifactorial nature of *in vitro* degradation pathways relevant for *in vivo* conditions.

### 3.4 *In vitro* assessment of cytocompatibility and biocompatibility of HA_ox_GM cryogels

**Fig. 4:**
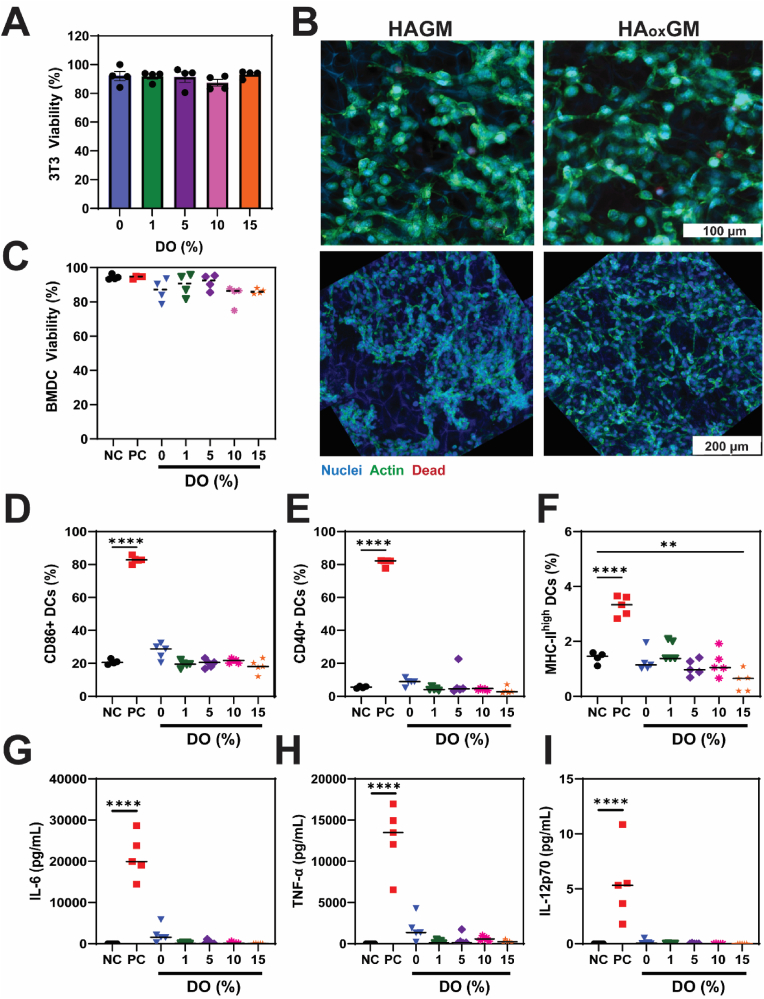
**HA_ox_GM cryogels are cytocompatible and do not trigger DC activation. A**) Cell viability (%) of NIH 3T3 cells after 24 h of culture within HA_ox_GM (DO = 0–15 %) cryogels. **B**) Confocal microscopy images of NIH 3T3 cells within HAGM and HA_ox_GM (DO 15 %), stained with DAPI (nuclear stain), cytoskeleton ActiStain^TM^, and FarRed viability stain. **C–F**) Fractions of live, CD86^+^, CD40^+^, and MHC-II^+^ BMDCs after 24 h of culture in various conditions: negative control (NC: cryogel-free RPMI media), positive control (PC: LPS-supplemented RPMI media), and HA_ox_GM (DO = 0–15 %) cryogels. **G–I**) Concentrations (pg/mL) of secreted proinflammatory cytokines: IL-6, TNF-α, and IL-12p70. Data are presented as mean ± SEM (n = 4–5). Statistical analysis was performed using one-way ANOVA and Tukey's post hoc test: ∗∗p < 0.01 and ∗∗∗∗p < 0.0001.

To ensure that the oxidation process does not impact the cytocompatibility of HA-based cryogels and their potential to support 3-dimensional (3D) cell culture, murine fibroblasts (NIH 3T3) were seeded onto HA_ox_GM-based cryogels. Following 24 h of incubation, the cells were stained and imaged using confocal microscopy to evaluate cell viability and interactions with the cryogels (Fig. 4A and B, Fig. S5). When compared to HAGM cryogel (DO = 0 %), which served as a baseline, the HA_ox_GM-based cryogels did not show a significant increase in the proportion of dead cells. Cell viability remained consistently above 80 % across all HA_ox_GM samples (DO = 1, 5, 10, and 15 %) (Fig. 4A). Furthermore, cytoskeleton staining revealed that the fibroblasts exhibited effective adhesion and elongation within the polymer network, suggesting good cytocompatibility (Fig. 4B).

To further assess their biocompatibility, we conducted a DC activation assay to evaluate the potential immunogenicity of HA_ox_GM-based cryogels. DCs, as key modulators of the adaptive immune response, act as antigen-presenting cells (APCs) that become activated and secrete proinflammatory cytokines in response to pathogens or toxic substances.

In this assay, DCs were incubated with HA_ox_GM-based cryogels (DO = 1, 5, 10, and 15 %). The results were compared to DCs exposed to HAGM cryogels (DO = 0 %), as well as to DCs exposed to cryogel-free (negative control) and LPS-containing (positive control) RPMI media. Positive and negative controls were used to establish the gating strategy, as depicted in Fig. S6. The data demonstrated that exposure to HA_ox_GM-based cryogels did not induce significant changes in DC viability and activation markers, including CD86, CD40, and MHC-II (Fig. 4C–F). Additionally, there was no notable increase in the production of proinflammatory cytokines IL-6, TNF-α, and IL12-p70 (Fig. 4G–I), indicating that HA_ox_GM cryogels do not exert a stimulatory effect on DCs. These findings confirm that HA_ox_GM-based cryogels are cytocompatible, possess favorable biocompatibility, and do not induce immunostimulation, suggesting their potential as a platform for various biomedical applications.

### 3.5 Biocompatibility and biodegradation of HA_ox_GM cryogels

**Fig. 5:**
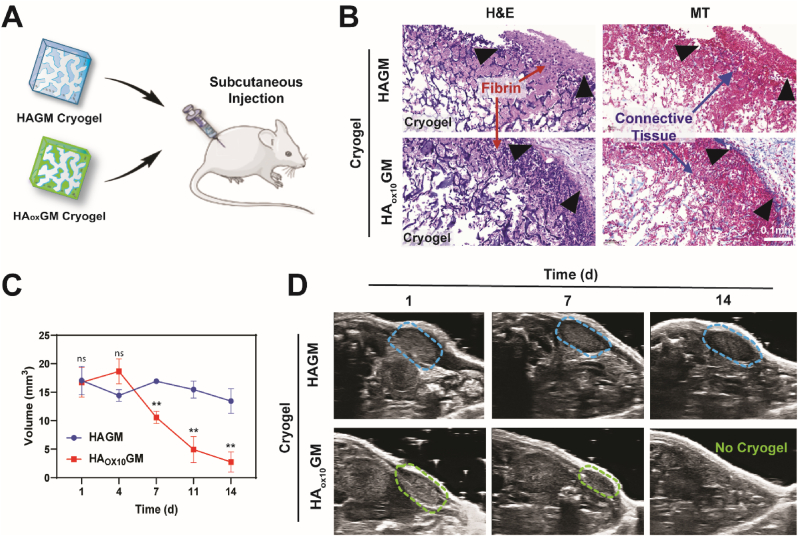
**HA_ox_GM cryogels are biocompatible and biodegradable *in vivo*. A**) Cryogels are injected subcutaneously into mouse flanks. **B**) H&E and MT staining of explanted HAGM and HA_ox_GM (DO 10 %) cryogels after 7 d. The black arrows indicate the boundary between the cryogel and the host tissue. **C–D**) Volume measurements of cryogels in mouse flanks over 14 d (C), calculated from 3D ultrasound imaging (D). Data are presented as mean ± SEM (n = 4–5). Statistical analysis was performed using a Welch's *t*-test to compare each timepoint: ns ≥ 0.05, ∗∗p < 0.01.

To further assess the biocompatibility and biodegradation of HA_ox_GM cryogels, we conducted an *in vivo* study using a murine model. HA_ox_GM (DO = 10 %) cryogels were selected for this study due to their relatively high degradation rate while still preserving syringe injectability and demonstrating cytocompatibility and *in vitro* biocompatibility. Both sterilized HA_ox_GM (DO = 10 %) and unoxidized HAGM (DO = 0 %) cryogels were injected subcutaneously into the dorsal flanks of C57BL/6 mice using 16G needles (Fig. 5A). After 7 d, the cryogels and surrounding tissues were explanted and subjected to histological analysis using H&E and MT staining (Fig. 5B). The histological evaluation revealed a mild inflammatory response, characterized by minimal leukocyte infiltration. The presence of fibrin and connective tissue indicated successful integration of the cryogels with the surrounding tissues. In addition, no differences in the inflammatory response were observed between HAGM and HA_ox_GM cryogels.

Notably, substantial bulk degradation of the HA_ox_GM cryogels was observed, as the cryogel constructs were no longer intact and showed signs of cleaved polymer fragments while the polymer walls were intact in the HAGM cryogels (Fig. S7). In a separate assessment, the 3D volume of the cryogels was monitored over a period of 14 d using ultrasound imaging (Fig. 5C). The HA_ox_GM cryogels exhibited a progressive reduction in volume over time, in contrast to the HAGM cryogels, which maintained their original shape and size throughout the observation period (Fig. 5D). Remarkably, HA_ox_GM cryogels were either entirely or nearly completely degraded within 14 d, a finding that contrasts sharply with the *in vitro* degradation prediction of approximately 140 d. This discrepancy highlights the limitations of *in vitro* models in accurately replicating the *in vivo* environment and, therefore, predicting the degradation behavior in the body.

### 3.6 Degradation of cryogels increases protein release *in vivo*

**Fig. 6:**
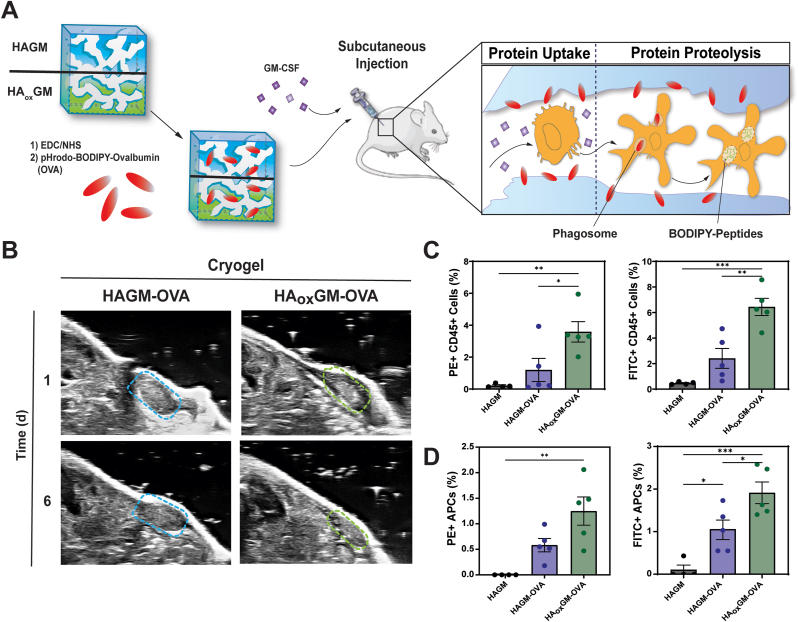
**Degradable cryogels enhance OVA delivery to infiltrating immune cells. A)** pHrodo-red and BODIPY labeled-OVA were conjugated to HAGM/HA_ox_GM cryogels using EDC/NHS chemistry and subcutaneously injected with mGM-CSF into the mouse flank. **B)** Ultrasound images taken 1 and 6 d after injection of OVA-loaded HAGM and HA_ox_GM cryogels. **C)** Fractions of PE^+^ (pHrodo-red) and FITC^+^ (BODIPY) CD45^+^ cells infiltrating the cryogels. **D)** Fractions of infiltrated PE^+^ (pHrodo-red) and FITC^+^ (BODIPY) MHC-II^+^ APCs. Data are presented as mean ± SEM (n = 4–5). Statistical analysis was performed using one-way ANOVA and Tukey's post hoc test: ∗p < 0.1, ∗∗p < 0.01, ∗∗∗p < 0.001, ∗∗∗∗p < 0.0001. (For interpretation of the references to colour in this figure legend, the reader is referred to the Web version of this article.)

To evaluate whether cryogel degradation could have a beneficial impact *in vivo*, we studied whether HA_ox_GM could enhance the release of a model protein, OVA. It has been previously demonstrated that biomaterials used to deliver molecules to infiltrating immune cells result in a peak in APCs around 1 week after implementation [52,53]. Since the HA_ox_GM cryogel (DO = 10 %) degrades within 2 weeks, we decided to use this formulation to investigate whether controlled degradation of cryogels could improve antigenic OVA delivery to infiltrating immune cells. First, it was confirmed that enhanced cryogel degradation facilitated protein release over a 2-week period *in vitro* (Fig. S8). Next, to assess phagocytosis, we used two dyes: pHrodo^TM^ Red (a pH-sensitive dye indicating protein localization inside the phagosome) and BODIPY^TM^ FL (quenched when OVA is heavily labeled but becomes strongly fluorescent after protein-induced hydrolysis into peptides). OVA was labeled with both fluorescent dyes and conjugated to HAGM and HA_ox_GM cryogels as previously described. The cryogels were sterilized and injected along mGM-CSF, an APC-recruiting cytokine, into the flanks of C57Bl/6 mice. Cryogel degradation was tracked using ultrasound imaging after 1 and 6 d (Fig. 6A and B). Captured ultrasound images indicated that, at 6 d after injection, HA_ox_GM-OVA cryogels had substantially decreased in size, whereas HAGM-OVA cryogels had maintained their volume. Mice were euthanized after 7 d, and cryogels were explanted and processed through a cell strainer to retrieve the infiltrated cell population. Cell suspensions were analyzed by flow cytometry to identify the fractions of protein (PE) and peptide (FITC) positive, CD45^+^ leukocytes, and MHC-II^+^ APCs (Fig. 6C–D, Fig. S9). Overall, our data shows that the release of OVA from degradable HA_ox_GM cryogels resulted in an over two-fold increase in the percentage of leukocytes and APCs positive for OVA and proteolyzed OVA, respectively, indicating that degradation improved OVA uptake and processing by infiltrating immune cells. These results demonstrate HA_ox_GM cryogels offer a significant utility in an *in vivo* context by providing a controllable degradation profile that is key to enhancing antigen release. This feature makes HA_ox_GM cryogels a highly promising vehicle for improving the targeted delivery of immunogenic agents to APCs, particularly in vaccine delivery systems where controlled release is critical for effective immune activation and response.

## 4 Discussion

Cryogelation of HAGM forms a highly crosslinked polymer network with a unique macrostructure, enabling exceptional compressibility and injectable constructs with shape memory properties. This interconnected macroporous network facilitates cellular infiltration, adhesion, and efficient diffusion of nutrients, oxygen, and pre-loaded molecules [48,54]. However, HA's susceptibility to degradation is hindered after modification with methacryloyl residues and cryopolymerization, resulting in a densely covalently crosslinked network containing polymethylmethacrylate segments, which are non-degradable. To overcome this limitation, we oxidized HA with NaIO_4_ before methacrylation and cryogelation, enhancing the degradability of cryogels while maintaining their mechanical integrity, macroporosity, syringe injectability, and biocompatibility.

Remarkably, HA_ox_GM (DO = 10 %) cryogels demonstrated almost complete degradation within 14 d *in vivo,* despite requiring 100 d to degrade *in vitro* under modeled physiological conditions. These findings highlight the limitations of *in vitro* models in accurately replicating *in vivo* degradation dynamics. Several factors likely contributed to the accelerated *in vivo* biodegradation, including local inflammation at the injection site, which may have lowered the pH and promoted acidic-catalyzed hydrolysis. *In vitro*, pH buffering systems rely on bicarbonate ions to maintain acid-base balance, thereby facilitating base-catalyzed hydrolysis of polysaccharides [9,42,55,56]. Our *in vivo* degradation results align with previous findings suggesting that, even under neutral or slightly acidic conditions, alkali-catalyzed β-elimination plays a significant role in depolymerization [40,42]. Additionally, increased enzymatic activity, oxidative depolymerization, and physical stress from mouse movement may further accelerate degradation [11,57]. Among these factors, physical stress could have had the most pronounced effect, as HA_ox_GM cryogels are mechanically weaker than HAGM cryogels. Although beyond the scope of this study, various strategies have been developed to enhance *in vitro* models for studying biomaterial degradation. While PBS is the most commonly used buffer to reflect the physiological pH of subcutaneous tissue, simulated body fluid (SBF) was developed to better mimic plasma and is frequently used to study drug release and degradability of delivery vehicles due to its improved ionic interactions [58,59]. In addition, devices such as the SC Injection Site Simulator (SCISSOR) have been designed to better model the release of small-molecule drugs from injected biomaterial depots [59]. However, existing *in vitro* methods are limited and cannot fully replicate the complex physiological mechanisms that facilitate biomaterial degradation.

For our *in vivo* studies, we selected the faster-degrading cryogels with the highest DOs while ensuring injectability and biocompatibility. However, while we cannot predict the behavior of cryogels with lower DOs *in vivo,* we anticipate that they would degrade more slowly and safely. In comparison, studies such as those by Chimpibul et al. suggest that varying DOs can significantly impact both degradation dynamics and tissue responses. In their study, oxidized cellulose scaffolds with a DO of 14.8 % exhibited low inflammation and fully degraded within 3 months, while those with a DO of 21.7 % degraded more rapidly within 1 month but caused higher inflammation and toxicity in rats [38]. These findings highlight the complexity of predicting both degradation rates and biocompatibility based on the DO. In contrast, our work confirmed good biocompatibility with HA_ox_GM (DO = 10 %) cryogels and observed a rapid degradation within 2 weeks. This difference is most likely due to the lack of enzymatic biodegradability of cellulose *in vivo*. Wang et al. used oxidized HA crosslinked with polyurethane (PU) to design biodegradable cryogels; however, they did not explore in detail how the DO could influence the degradation rate, focusing instead on optimizing the HA-to-PU ratio [60]. They reported a DO of 45 %, with a 30 % mass loss after 1 month of degradation in PBS. Additionally, they reported that the use of PU was required to maintain robust mechanical properties. These results underscore the versatility of the oxidation strategy, which can be tailored for specific systems by adjusting factors such as the choice of polymer and the DO.

It was hypothesized that the rapid degradation of cryogels within a couple of weeks could enhance the uptake of the immunogenic antigen, OVA, by immune cells migrating to the cryogel after subcutaneous injection in mice. Indeed, cryogel-infiltrating APCs exhibited a more than two-fold increase in the percentage of cells that phagocytosed OVA in OVA-containing HA_ox_GM (DO = 10 %) cryogels compared to OVA-containing HAGM cryogels. Additionally, the frequency of APCs positive for FITC-labeled peptides, formed after protease breakdown of OVA, was higher from OVA-containing HA_ox_GM (DO = 10 %) cryogels, suggesting that more OVA was processed by APCs, likely due to better release of OVA from HA_ox_GM cryogels compared to HAGM cryogels. These results align with the findings of Luo et al., which demonstrated that controlled degradation of cryogels can enhance antigen release, emphasizing that tailoring degradation processes can yield favorable outcomes for immune cell stimulation [61]. The strategic choice of using HA as the building block for cryogels may have facilitated the uptake of HA_ox_GM-OVA fragments by APCs through interactions with HA-binding CD44 receptors [62]. HA_ox_GM was also found to have a lower molecular weight compared to HAGM, which may have influenced the phagocytosis and subsequent processing of HA_ox_GM-OVA fragments. Dalla Pieta et al. demonstrated that conjugating OVA to various molecular weight HA influenced the adaptive immune response to the vaccine, with HA serving as an adjuvant to boost DC maturation, antibody production, and OVA-specific T cell responses, although they did not evaluate the effect on DC uptake [63]. In addition, cryogel degradation may have also allowed better access for APCs to interact with OVA. Recently, Meany et al. showed that adjuvanted hydrogel depots loaded with mCherry-labeled mRNA lipid nanoparticles had 1–3 % mCherry^+^ infiltrating leukocytes after 7 d [53]. Similarly, HAGM-OVA cryogels showed comparable percentages of PE^+^ and FITC^+^ leukocytes, but HA_ox_GM-OVA cryogels significantly improved upon this, highlighting their potential to advance current biomaterial-based delivery platforms. Future studies should further investigate the relationship between cryogel degradation rate and protein delivery to APCs to better understand how degradation influences protein uptake and immune activation.

In addition to their potential use in drug delivery, degradable HA_ox_GM cryogels have potential applications in wound healing, tissue engineering, and 3D tumor modeling. For these applications, HA_ox_GM cryogels could be functionalized to carry specific bioactive molecules, such as cell-adhesive moieties (e.g., peptides, proteins) to promote cell-matrix interactions. These biomolecules can be chemically conjugated via carbodiimide reactions or click chemistry [64,65]. However, one advantage of having pendant aldehyde residues on oxidized HA is the ability to couple biomolecules via reductive amination between the carbonyl and an amine, providing an alternative strategy [66,67]. However, it has been reported that reducing the aldehyde group influences the structure and stability of the polymer, limiting its susceptibility to hydrolysis and resulting in degradation profiles similar to those of the unmodified polymer itself [40]. Therefore, further research is needed to identify an optimal balance between the degree of aldehyde reactivity and maintaining controlled cryogel degradation.

Additionally, the degradation rate of these cryogels could be tailored to match tissue regeneration rates *in vivo*. For example, Thai et al. engineered poly(ethylene) glycol hydrogels with degradable matrix metalloproteinase crosslinks to create hydrogels with tunable degradation rates, enhancing the delivery and therapeutic potential of cell spheroids for wound healing [68]. Controlled degradation of the biomaterial improved cellular metabolism and the secretion of proangiogenic cytokines, thereby increasing the efficacy of the hydrogels in promoting wound healing [68]. This work supports the potential of controlled gel system degradation for enhancing therapeutic tissue regeneration. Further investigation is also required to better understand the effects of degradation byproducts, including their reabsorption or clearance from the body, to evaluate potential complications and long-term effects. While we did not observe increased inflammation during cryogel degradation, low molecular weight HA fragments are known to be more immunogenic than high molecular weight HA, which could modulate biological responses and impact the overall biocompatibility of the biomaterial [63].

## 5 Conclusion

To our knowledge, this is the first report to utilize an oxidized polymer to engineer injectable cryogels specifically for assessing their biodegradation in both *in vitro* and *in vivo* settings. Our study shows that HA_ox_GM cryogels retain the favorable intrinsic properties of cryogels, including minimally invasive delivery and an ideal macroporous structure while providing precise control over degradation through various mechanisms. The cytocompatibility of HA_ox_GM cryogels was confirmed *in vitro*, and their biocompatibility was validated *in vivo*, with results demonstrating bulk degradation without inducing local inflammation in mice. As a proof of concept, our *in vivo* degradation study also validated the utility of controlled biodegradation of HA_ox_GM cryogels, with data showing that they significantly enhanced the uptake of fluorescently-labeled OVA by infiltrating immune cells (e.g., APCs), highlighting their potential as an effective platform for improved delivery and immunomodulation. These findings emphasize the potential of HA_ox_GM cryogels as versatile, biocompatible materials for biomedical applications, particularly in contexts where controlled degradation is a critical feature.

## CRediT authorship contribution statement

**Alexandra Nukovic:** Writing – review & editing, Writing – original draft, Visualization, Validation, Methodology, Investigation, Data curation, Conceptualization. **Mohammad Hamrangsekachaee:** Validation, Methodology, Investigation, Conceptualization. **Mahalakshmi Rajkumar:** Validation, Methodology, Investigation. **Gwyneth Wong:** Validation, Investigation. **Emily R. Tressler:** Validation, Investigation. **Sara M. Hashmi:** Validation, Methodology. **Stephen M. Hatfield:** Writing – review & editing, Writing – original draft, Supervision, Funding acquisition. **Sidi A. Bencherif:** Writing – review & editing, Writing – original draft, Validation, Supervision, Project administration, Funding acquisition, Conceptualization.

## Funding

S.A.B. and S.M.H. gratefully acknowledges the financial support from the 10.13039/100000002National Institutes of Health (NIH, 1R01EB027705). S.A.B. also acknowledges funding from the 10.13039/100000001National Science Foundation (NSF CAREER, DMR 1847843) and the *Chaire d’Excellence de Normandie*.

## Declaration of competing interest

The authors declare that they have no known competing financial interests or personal relationships that could have appeared to influence the work reported in this paper.

## Data Availability

Data will be made available on request.
