# Supplementary material for: Polymer oxidation: A strategy for the controlled degradation of injectable cryogels
Source: Mater Today Bio. 2025 Apr 8;32:101743. doi: 10.1016/j.mtbio.2025.101743 (PMC12059720; doi:10.1016/j.mtbio.2025.101743)
Supplement: Multimedia component 1 [file mmc1.pdf]

Supplementary Information

Polymer oxidation: A strategy for the controlled degradation of injectable cryogels

Alexandra Nukovic[1], Mohammad Hamrangsekachaee[1], Mahalakshmi Rajkumar[2], Gwyneth Wong[3], Emily Tressler[2], Sara M. Hashmi[1], Stephen M. Hatfield[1,3,4,*], and Sidi A. Bencherif[1,2,5,6*]

1. Department of Chemical Engineering, Northeastern University, Boston, MA, 02115, USA
2. Department of Bioengineering, Northeastern University, Boston, MA, 02115, USA
3. Department of Biology, Northeastern University, Boston, MA, 02115, USA
4. New England Inflammation and Tissue Protection Institute, Department of Pharmaceutical Sciences, Northeastern University, Boston, MA, 02115, USA
5. Harvard John A. Paulson School of Engineering and Applied Sciences, Harvard University, Cambridge, MA, 02138, USA
6. University Rouen Normandie, CNRS, PBS UMR 6270, F-76000, Rouen, France

* Corresponding authors:

Sidi A. Bencherif (s.bencherif@northeastern.edu)

Stephen M. Hatfield (s.hatfield@northeastern.edu)

Table S1. $NaIO_4$ addition for targeted HA oxidation. Amount of $NaIO_4$ added to HA solution (200 mL) to achieve a target degree of oxidation (DO) varying between 5% and 40 %.

Theoretical DO (%)	$NaIO_4$ (mg)
5	26.67
10	53.34
20	106.66
40	213.3

Table S2. Molar mass moments and polydispersity. Number average molecular weight (M_n), peak molecular weight (M_p), weight average molecular weight (M_w), Z-average molecular weight (M_z), and polydispersity (PDI) for HAGM, HA_{ox} (DO = 1%), and $HA_{ox}GM$ (DO = 1%). The reported values represent the average ± one standard deviation of triplicate measurements.

Sample	M_n (kDa)	M_p (kDa)	M_w (kDa)	M_z (kDa)	PDI
HAGM	370 ± 33	526 ± 13	759 ± 35	1,370 ± 146	2.06 ± 0.11
HA_{ox}	83 ± 4	133 ± 2	144 ± 4	202 ± 6	1.74 ± 0.09
$HA_{ox}GM$	43 ± 5	64 ± 4	85 ± 6	582 ± 416	2.02 ± 0.33

Table S3. Estimated cryogel injection shear rates. Shear rate (1/s) during syringe injection was estimated based on needle radius (mm), needle length (mm), and injection time (s).

DO (%)	Plunge Time (s)	Shear Rate (1/s)
0	1	31
1	1.5	21
5	1	31
10	0.75	42
15	0.75	42

Table S4. Swelling ratio of cryogels over time. The swelling ratio (Q_M) of HAGM and $HA_{ox15}GM$ cryogels was measured after 5 min (0 h) and 24 h in PBS.

Time (h)	HAGM Q_M	$HA_{ox15}GM$ Q_M
0	45.6 ± 3.8	40.2 ±1.6
24	49.5 ± 4.4	35.8 ± 0.2

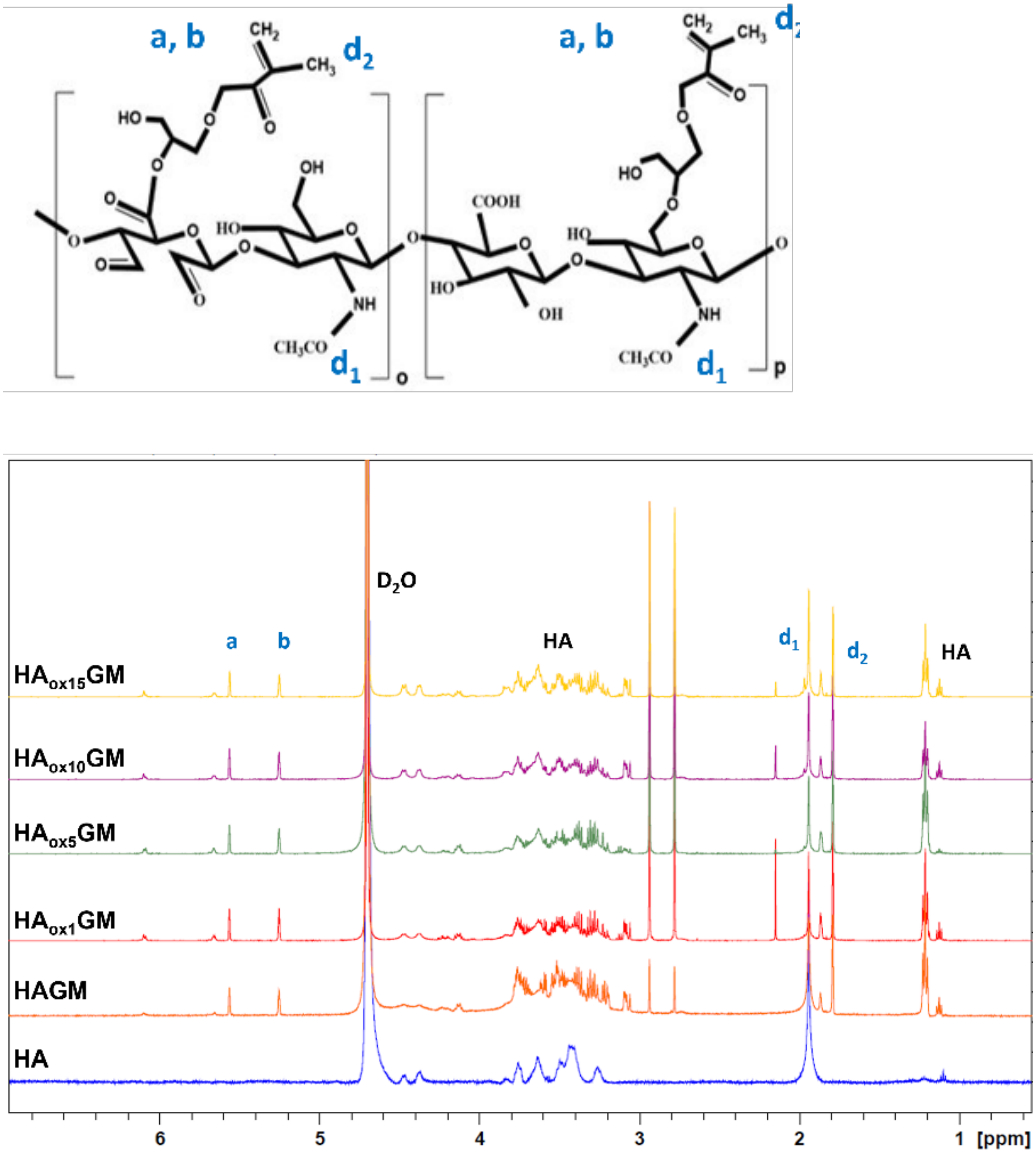

Figure S1. ^{1}H NMR characterization of HA and its derivatives. ^{1}H NMR spectra of HA, HAGM, and $HA_{ox}GM$ (DO = 1–15%) in D_2O with corresponding peaks of HA and methacryloyl residues.

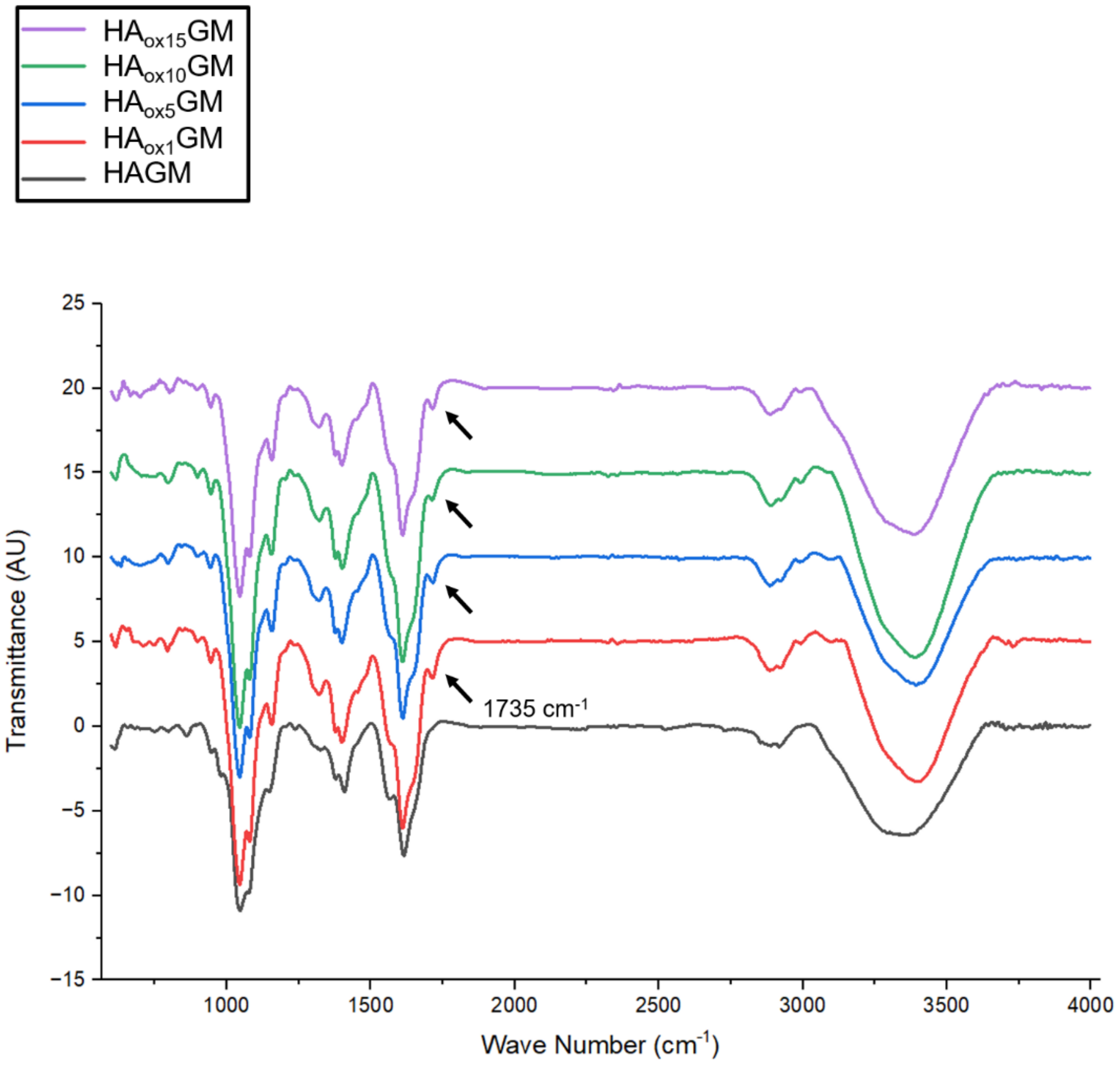

Figure S2. ATR-FTIR of HA$_{ox}$GM. ATR-FTIR spectra of HAGM and HA$_{ox}$GM at various DOs (1–15%). The spectra show the introduction of characteristic aldehyde peaks at wave number 1735 cm^{-1}, corresponding to the presence of aldehyde groups in oxidized HA.

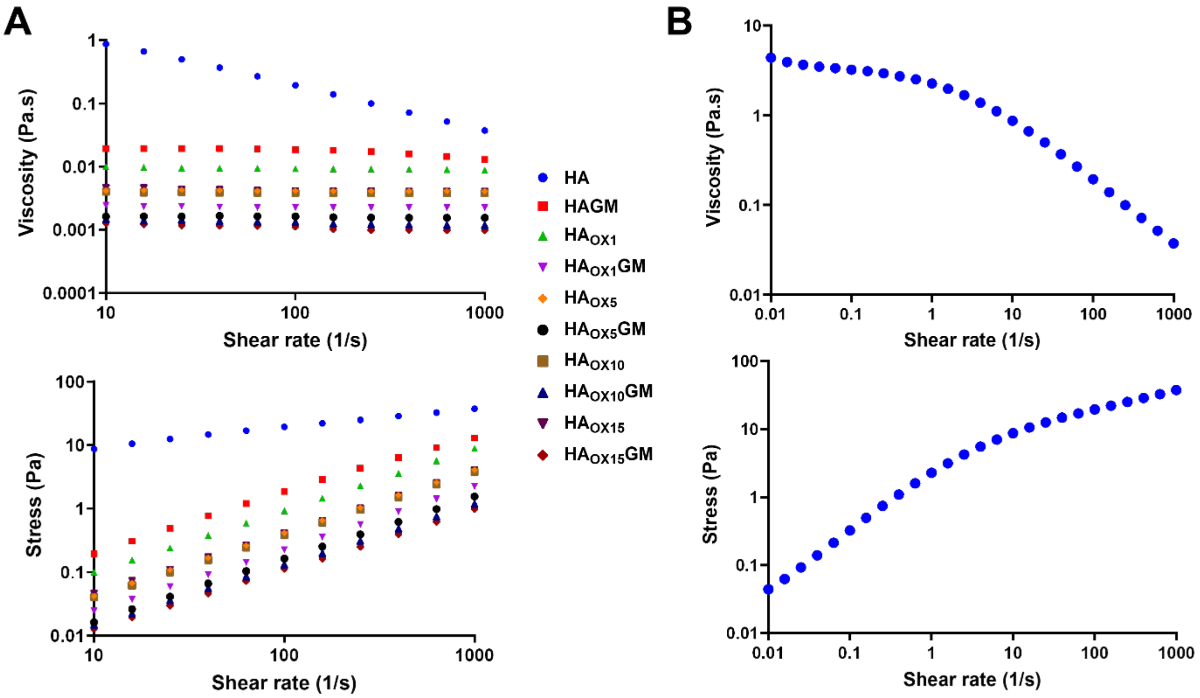

Figure S3. Rheological measurements of HA and its derivatives. A) Viscosity (Pa.s) and stress (Pa) of polymer solutions (0.5 % w/v) of HA, HAGM, HA_{ox}, and $HA_{ox}GM$ in diH_2O at varying shear rates (1/s). **B)** Viscosity (Pa.s) and stress (Pa) as a function of shear rate (1/s) of HA. Non-modified HA exhibits a non-Newtonian behavior, characterized by a decreasing viscosity under increasing shear rate. In contrast, the modified HA variants (HA_{ox}, HAGM, and $HA_{ox}GM$) display Newtonian behavior, indicating a constant viscosity with increasing shear rate.

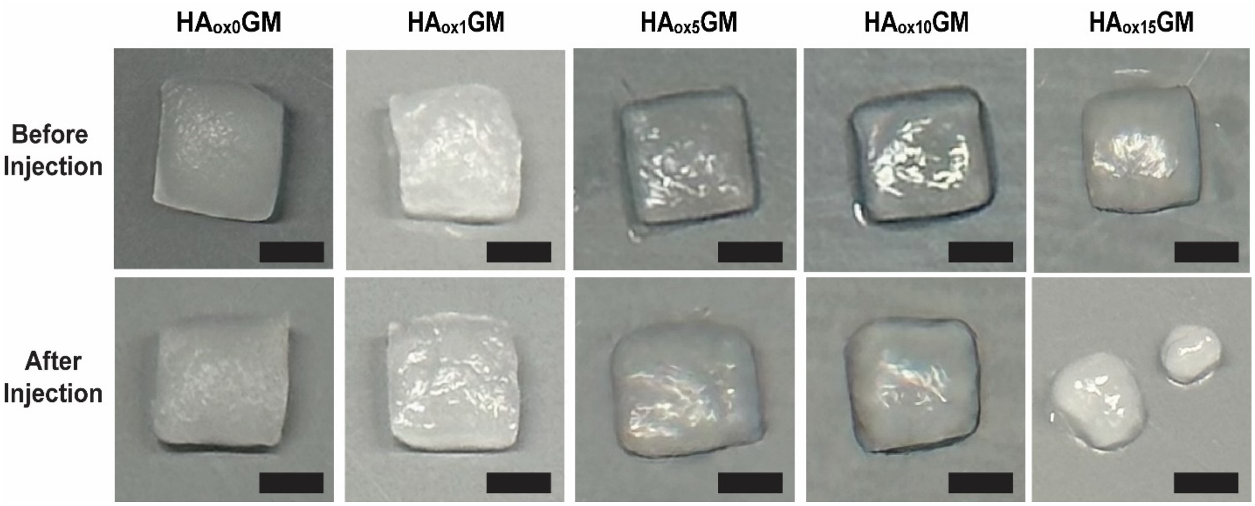

Figure S4. Shape memory of cryogels. Representative photos of cryogels with varying DOs (0–15%) captured before and after injection through a 16G hypodermic needle, demonstrating their ability to recover their original shape. Scale bar = 2 mm.

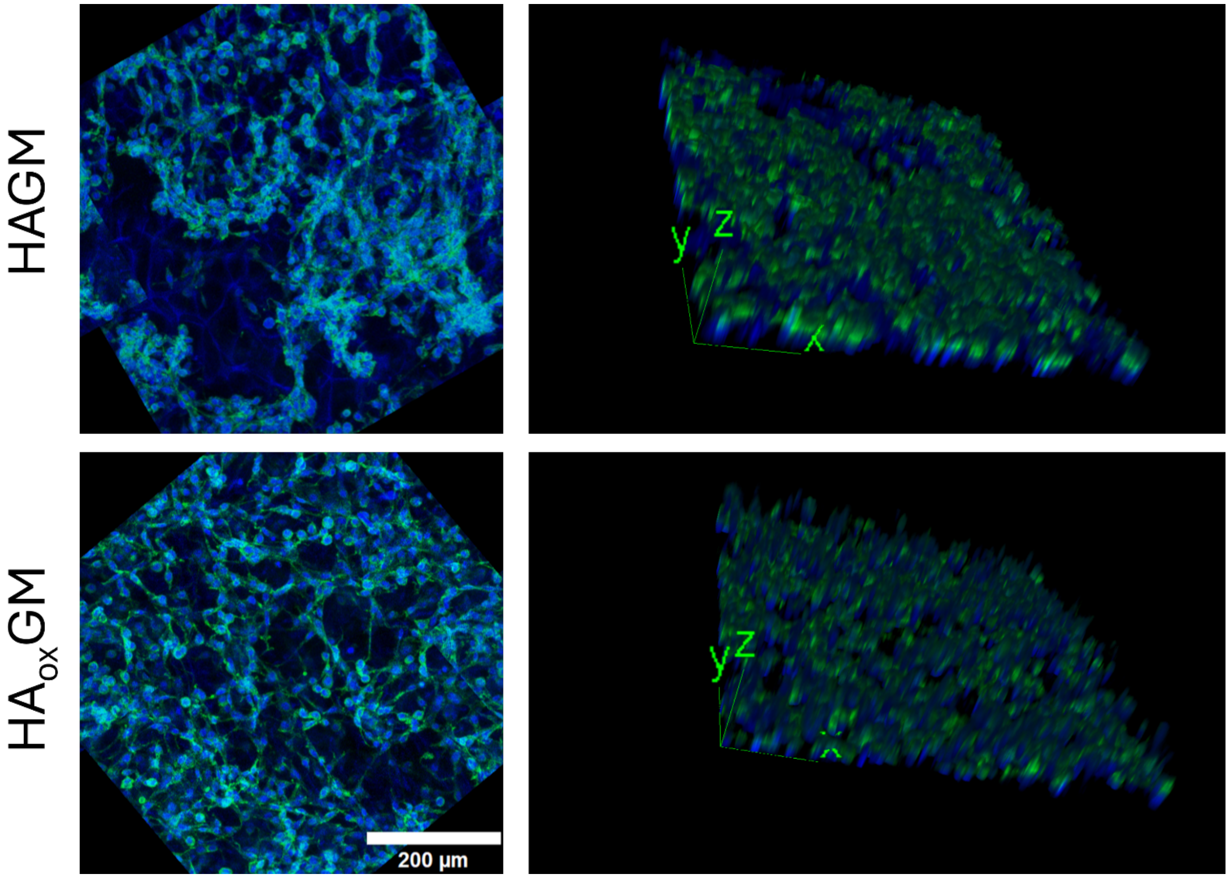

Figure S5. Three-dimensional (3D) images of cell-seeded cryogels. A selected region within the cell-seeded cryogel was visualized in the Z-axis view (left) and the 3D reconstruction (right) of confocal microscopy z-stacks, showing adherent and elongated NIH 3T3 cells homogeneously distributed within RGD-containing cuboidal HAGM and $HA_{ox}GM$ (DO = 15%) cryogels, stained with DAPI (nuclear stain) and ActiStain™ (cytoskeleton).

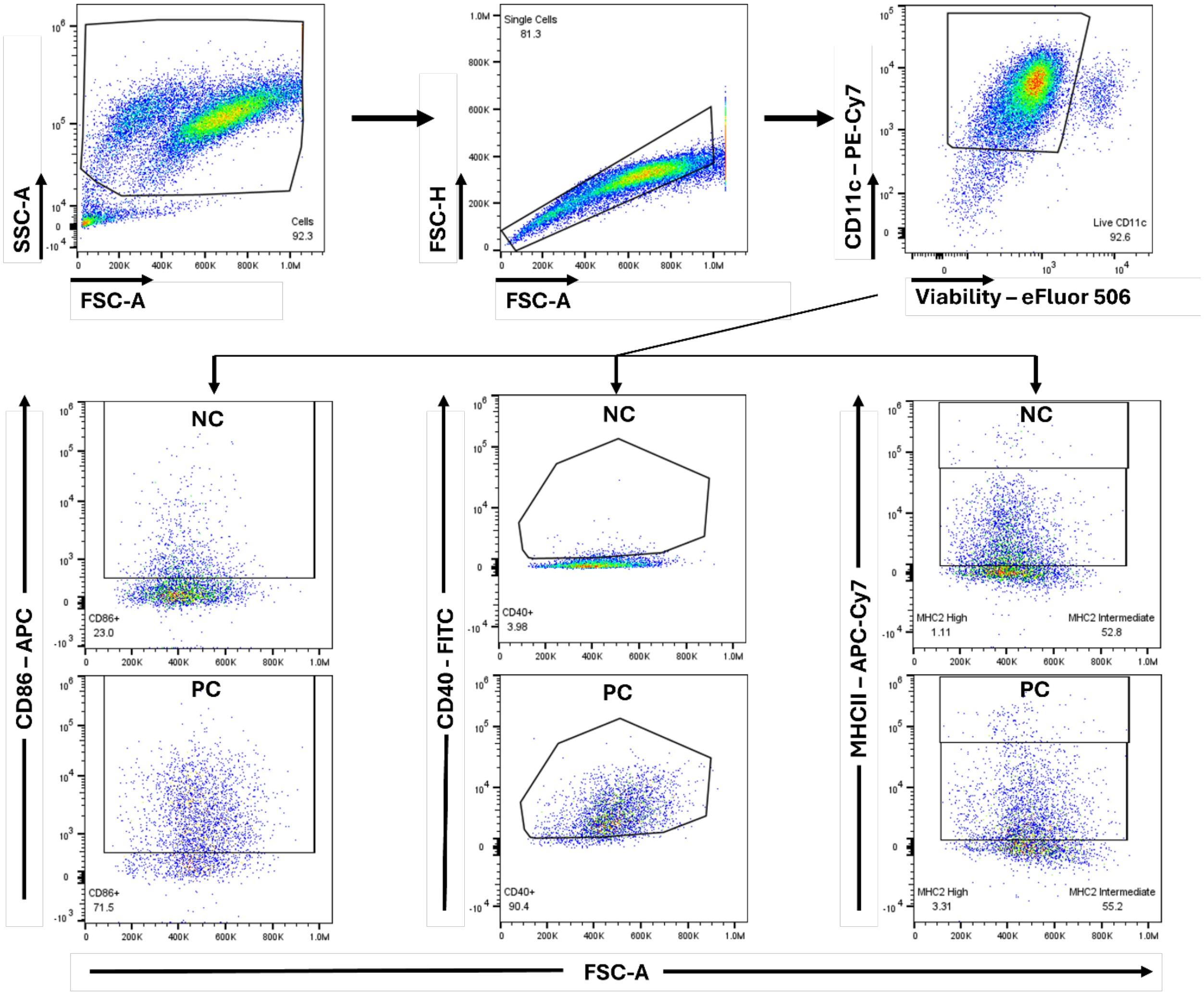

Figure S6. Analysis of bone marrow-derived dendritic cells (BMDCs) by flow cytometry. The gating strategy was used to identify live CD11c+ dendritic cells (DCs) expressing CD86, CD40, and MHCII. The negative control (NC) represents DCs cultured in complete media, while the positive control (PC) corresponds to LPS-stimulated DCs.

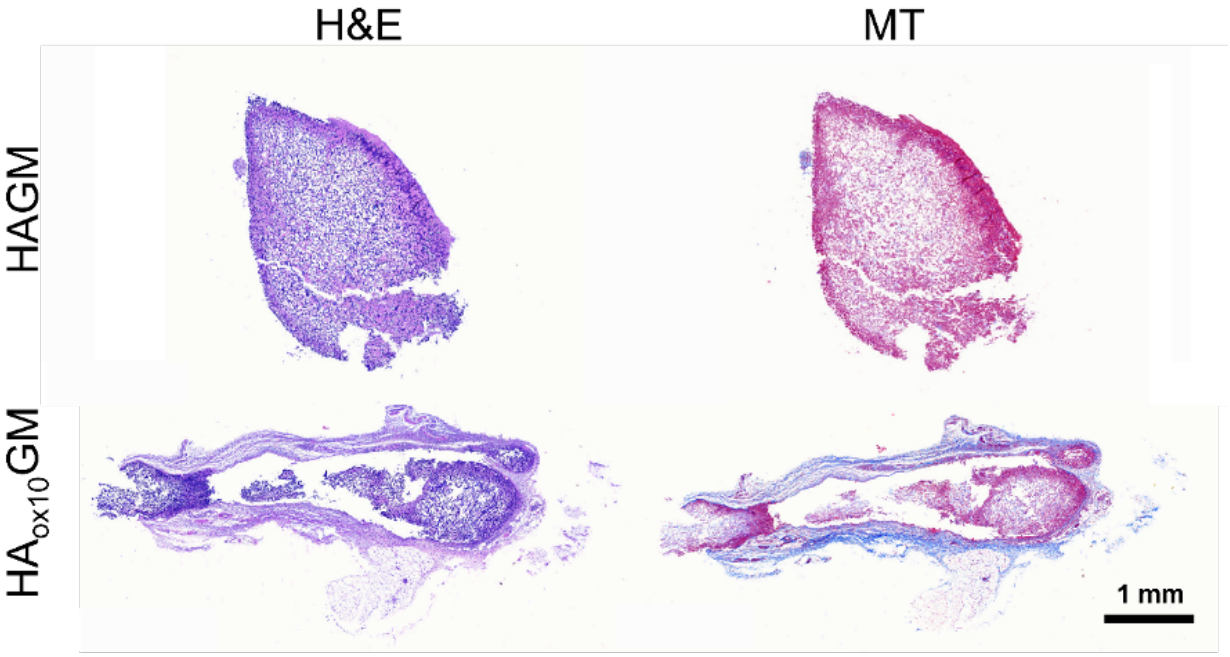

Figure S7. Biocompatibility of cryogels. Histological analysis of cryogels (HAGM and $HA_{ox10}GM$) explanted 7 d after subcutaneous injection in mice. Hematoxylin and eosin (H&E) and Masson's trichrome (MT) staining were performed. Histological images are representative of n = 4 samples per condition.

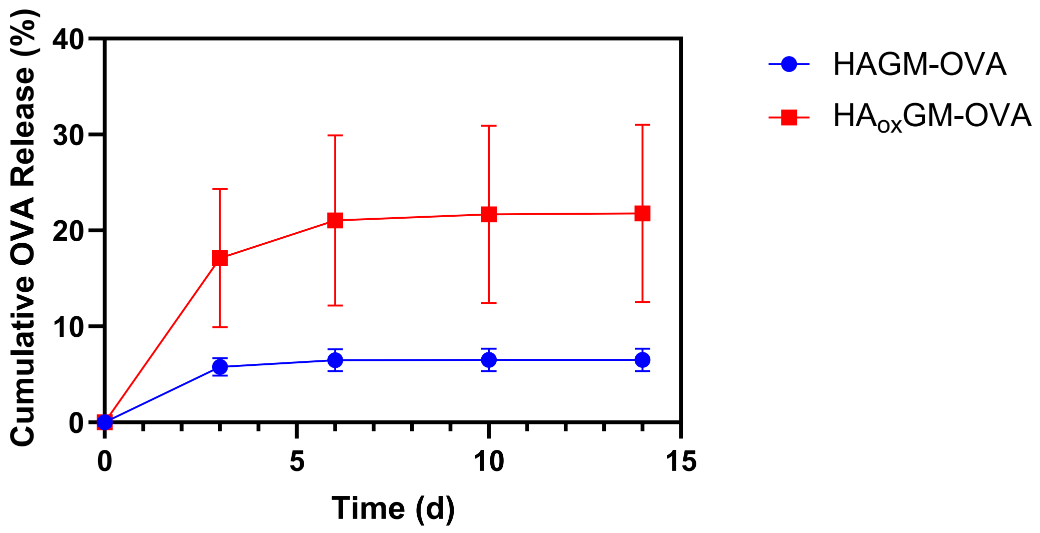

Figure S8. Release profiles of OVA from cryogels. OVA-conjugated HAGM and $HA_{ox10}GM$ cryogels were incubated in PBS at 4 °C under orbital shaking. At various time points over a 2-week period, the supernatant was collected, and OVA release was quantified using a Micro-BCA assay.

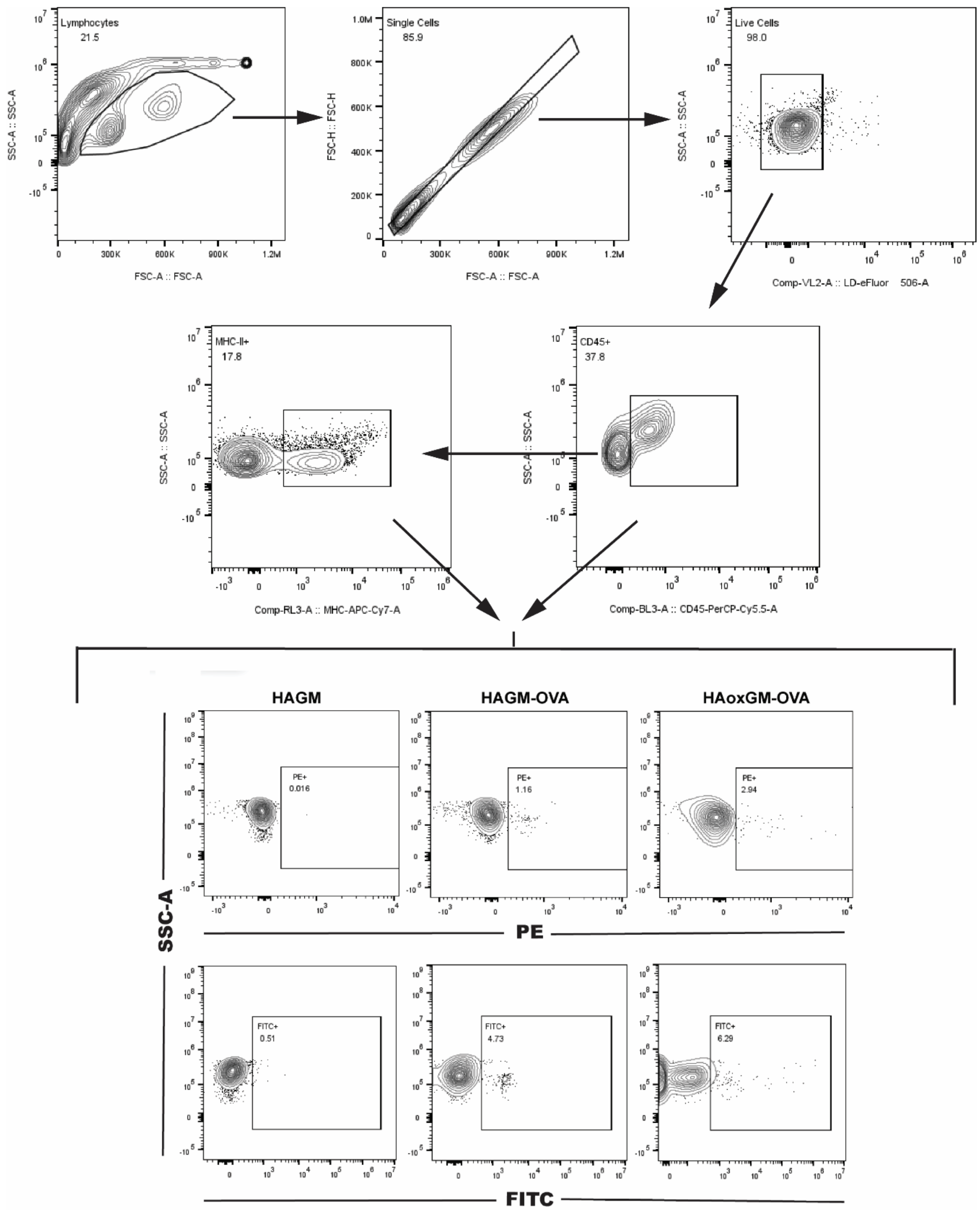

Figure S9. Analysis of infiltrating immune cells by flow cytometry. Gating strategy for PE+ FITC+ live CD45+ and MHC-II+ cells collected from HAGM, HAGM-OVA, and HA_{ox}GM-OVA cryogels explanted 7 d after subcutaneous injection in mice.
